# YOLOv5 with ConvMixer Prediction Heads for Precise Object Detection in Drone Imagery

**DOI:** 10.3390/s22218424

**Published:** 2022-11-02

**Authors:** Ranjai Baidya, Heon Jeong

**Affiliations:** 1 Pattern Recognition and Machine Learning Laboratory, Gachon University, Seongnam 13120, Korea; 2 Department of Fire Service Administration, Chodang University, Muan 58530, Korea

**Keywords:** object detection, YOLOv5, ConvMixer, UAV imagery

## Abstract

The potency of object detection techniques using Unmanned Aerial Vehicles (UAVs) is unprecedented due to their mobility. This potency has stimulated the use of UAVs with object detection functionality in numerous crucial real-life applications. Additionally, more efficient and accurate object detection techniques are being researched and developed for usage in UAV applications. However, object detection in UAVs presents challenges that are not common to general object detection. First, as UAVs fly at varying altitudes, the objects imaged via UAVs vary vastly in size, making the task at hand more challenging. Second due to the motion of the UAVs, there could be a presence of blur in the captured images. To deal with these challenges, we present a You Only Look Once v5 (YOLOv5)-like architecture with ConvMixers in its prediction heads and an additional prediction head to deal with minutely-small objects. The proposed architecture has been trained and tested on the VisDrone 2021 dataset, and the acquired results are comparable with the existing state-of-the-art methods.

## 1. Introduction

Drones and Unmanned Aerial Vehicles (UAVs) are equipped these days with state-of-the-art computer vision technologies. The UAVs equipped with such technologies have been used comprehensively in a wide range of application areas including but not limited to security surveillance [1,2,3], aerial filming [4], high-speed delivery [5], wild life conservation [6,7,8,9], and agriculture [10]. Throughout the mentioned use case areas, the UAVs are aware of the surrounding environment and respond to the changes in the environment. The awareness of the UAVs may come in various ways; one commonly used way is by using object detection in drone-captured images. Hence, object detection in general and object detection in UAV applications has attracted significant interest from researchers.

The field of computer vision is evolving rapidly, with a significant focus on obtaining more accurate results and speed for real-time applications. This evolution has been supported by better and more powerful computing resources and abundant high-quality labeled data (e.g., ImageNet [11] and COCO [12]). The transition from traditional image processing techniques to Deep Convolutional Neural Networks (CNNs) (e.g., ResNet [13] and DenseNet [14]) and recently to attention-based transformers (e.g., Vision Transformer [15], Swin Transformer [16]) has been possible due to the availability of better resources. With this, the field of computer vision has also habituated more complex methods for other generic computer vision applications such as segmentation and action recognition. Similarly, there has been rapid development in the general object detection domain. However, the progress has yet to be replicated for object detection with images taken from UAVs. This lag is primarily due to issues specific to UAV imagery. Due to variations in the deployment environment and computing resource constraints, multiple challenges arise in object detection in UAV applications. These challenges include: (i) Variations in object scale due to variations in altitude of flight of the UAVs, (ii) images taken from higher altitude mostly contain objects at high density and the size of the objects are very small, and (iii) a wide distribution of the background environment [17]. To strengthen the ability of drones to sense the deployment environment, it is necessary that these challenges are considered during the system design phase.

The YOLO (You Only Look Once) model series has been influential in the development of object detection [18,19,20,21]. These models have always performed well in obtaining state-of-the-art results in general object detection. However, these models are only partially suited for object detection in UAV images. In this paper, we modify the existing YOLOv5 model to address the object detection problem in UAV images. To that extent, we look at utilizing the ConvMixer architecture at the head of a YOLOv5-like architecture with an additional prediction head. Adding ConvMixer to the prediction head helps find the spatial and channel-wise relationships in the features extracted by the body and delivered by the neck of the architecture. In ConvMixers, these spatial and channel-wise relationships are extracted by depthwise and pointwise convolutions, respectively. Pointwise convolutions also enhance the capability of the prediction heads to obtain better detections in the case of minutely small objects, as it deals with individual data-point-level information. Furthermore, the ConvMixer architecture is straightforward as it only utilizes simple convolutions alongside batch normalization layers. In addition, the ConvMixer architecture maintains the input structure throughout the number of used layers, making it suitable to be used in the head of object-detection architectures. We use the CSPDarknet53 and path aggregation network (PANet) as the backbone and the neck, respectively, as in the original YOLOv5 architecture. We also utilize proven methods such as Convolutional Block Attention Module (CBAM) in a similar way as in TPHYOLOv5 to generate channel-wise and spatial attention. The suggested model performed better than the YOLOv5 and TPH-YOLOv5 models when tested on the VisDrone 2021 dataset.

The contributions of this paper can be listed as follows:

- We propose a single-stage object detection model designed to address the challenges set forth by UAV images during object detection.
- We integrate ConvMixer into the prediction heads of the YOLOv5 for better detection of objects in UAV imagery settings.
- Our proposed method performs better than the baseline YOLOv5 and TPH-YOLOv5 models.

## 2. Related Work

### 2.1. Object Detection

Traditional methods for object detection utilize feature extraction methods such as Histogram of Oriented Gradients (HOG) [22] or Scale Invariant Feature Transform (SIFT) [23]. For the features extracted using these methods to be helpful for any specific use case scenarios, they require a substantial amount of human input and time, which is inconvenient. The current scenario of the field of object detection is different. The field of object detection has prospered with the emergence and development of CNN architectures, which learn the appropriate features from the unprocessed data for performing the respective tasks at hand. One reason for the rapid development of these architectures is the availability of powerful hardware such as GPUs and TPUs, which make it favorable for these complex algorithms to be utilized.The currently available modern object detection pipelines can be divided into two categories based on their usage of pre-defined sliding windows: anchor-based and anchor-free object-detection architectures.

The anchor-based methods sample the boxes into separate bins and rectify the boxes of objects. On the other hand, anchor-free methods do not deal with the computations related to anchor boxes and use some substitute methods. Fully Convolutional One-Stage (FCOS) [24] is an example of an anchor-free method that performs the prediction in per-pixel fashion. Unlike other object detection methods, FCOS depends on the number of objects to be detected and center-ness threshold rather than the Intersection of Union (IoU). Feature Selective Anchor Free Module (FSAF) [25] is another anchor-free method where multi-level anchor-free branches are used on a pyramid structure, and the online feature selection is used. An anchor-free branch is attached to every level of the pyramid, so the box encoding and decoding are possible in an anchor-free manner at an arbitrary level. Generalized focal loss (GFL) v1 [26] unifies the quality estimation and the class prediction vector and creates a joint representation. Additionally, they use a vector representation for the arbitrary distribution of box locations. The second version of GFL [27] utilizes GFLv1 and considers the correlation between distribution statistics and the real localization quality to create a lightweight Distribution-Guided Quality Predictor (DGQP), resulting in GFLv2. YOLOX [28] switches the YOLO detector into an anchor-free detector and implements other proven techniques such as a decoupled head [24,29,30] and the label assignment strategy SimOTA.

The anchor-based methods can further be classified based on the steps required for the localization and classification of the objects into multi-stage detectors and single-stage detectors. The multi-stage detection frameworks use a tailored search algorithm in the first stage to distinguish the prospective areas for object detection. In the second stage, a feature-extracting architecture extracts features from the candidate areas. Then, a classifier is used to categorize those areas into object detection classes. Region-CNN (RCNN) [31] is an example of a regular multi-stage object detection algorithm. RCNN has the drawback of only being able to perform object detection on images of a fixed size. To overcome the drawback of RCNN, the Spatial Pyramid Pooling (SPP) Network [32] utilizes spatial pyramid pooling, a combination of multiple pooling layers of different kernel sizes. The SPP layer is used before the fully connected (FC) layer, which flattens the output of the multiple pooling kernels and sends it to the FC layer. Taking things to the next level, Fast RCNN [33] introduced ROI pooling, which shares the computations of all of the proposals rather than independently performing individual calculations for each proposal. In addition, the prospective regions suggested by the first stage of Fast RCNN have considerably less overlap than RCNN. Another multistage detector, Faster-RCNN [34], introduces a region proposal network (RPN) that generates proposals at various scales and aspect ratios. The model is also capable of knowing where to look for the objects. Furthermore, the work introduces the concept of anchor boxes, which prevents the usage of pyramids of images, i.e., multiple instances of the same images at different scales. Finally, Cascade-RCNN [35] utilizes multiple heads, where the samples from the output of the heads are filtered using a threshold more prominent than that of the previous head and passed on for more precise scaling and offsets, which tends to make the predictions closer to the labels.

The single-stage object detection models are end-to-end models used to give the bounding boxes and the class probabilities at once. These models are faster and computationally less expensive than multi-stage object detection models. Single-Shot multibox Detector (SSD) [36] is a VGG-based object detection model that can perform object localization and classification in a single forward pass. It utilizes the bounding box regression technique inspired by Multibox [37]. In another work, RetinaNet [29], the category imbalance problem of single-stage detectors is addressed. It utilizes focal loss to address the issue. You Only Look Once (YOLO) [18] was another step toward pursuing speed in object detection. YOLOv2 [19] utilizes Darknet-19 instead of the GoogleNet used in the original YOLO. YOLOv3 [20] upgraded Darknet-19 to Darknet-53, which consisted of the multiscale framework and the concept of residual connections from ResNet [13]. YOLOv4 [21], although not from the authors of the original YOLO series, took the YOLOV3 architecture a step further by utilizing the Cross Stage Partial Network (CSPNet) structure [38] with the Darknet-53 framework to form the backbone of the network. Furthermore, it used Complete Intersection of Union loss (CIoU) [39] and the Mish activation function [40] to enhance the object detection performance. Finally, YOLOv5 adopts all of the abovementioned architectures and provides a series of architectures with varying inference speed, accuracy, and FLOPs.

### 2.2. Object Detection on UAV-Captured Images

In many aspects, the object detection scenario in UAV-captured images is distinct from the general object detection scenarios. First, the shapes and sizes of the objects detected vary widely in UAV-captured images. Similarly, there is always a possibility that the number of objects to be detected in UAV scenarios is high. Furthermore, there is also a limitation regarding computing resources in UAVs. Due to these reasons, the progress of object detection in generic scenarios (e.g,. PASCAL VOC [41] and COCO) has yet to be replicated in object detection using UAV images. Many works have been undertaken focusing on the object’s size and mainly employ coarse-to-fine frameworks: Peele [42], ClutDet [43], and DMNet [44]. TPHYOLOv5 has added one additional prediction head to the existing prediction heads of YOLOv5. Furthermore, all of the prediction heads are transformer-based [17]. Another method called M-CenterNet, which uses multiple center points, has also been suggested to deal with tiny objects in aerial footage [45]. To implement a more lightweight version of YOLOv3 in the object detection scenario of UAVs, a slimmer version YOLOV3 called SlimYOLOV3 was also presented, which used sparsity training, pruning, and fine-tuning for slimming [46]. RRNet [47] incorporates anchor-free detectors with a re-regression module to fabricate a hybrid detector specifically designed for the scenario of UAV imagery.

## 3. Methods

In this section, we discuss the used models such as YOLOv5, TPHYOLOv5, Transformers, ConvMixer [48], and Convolutional Block Attention Module CBAM [49] before discussing the detailed overall suggested architecture. Additionally, we also compare Transformers and ConvMixers.

### 3.1. Overview of YOLOv5

You Only Look Once (YOLO) is a famous line of single-stage objection detection architectures. YOLO was the first object-detection architecture capable of finding the bounding boxes of objects and classifying the labels in an end-to-end network. This original YOLO framework and its succeeding YOLOv2 are based on the Darknet architecture.

YOLOv5, while not from the original authors of YOLO, is currently one of the most famous architectures used for object detection. There are five different architectures of different sizes suggested under the YOLOv5 name tag: YOLOv5n, YOLOv5s, YOLOv5m, YOLOv5l, and YOLOv5x. Each model utilizes the CSPDarknet53 architecture and the SPP layer as the backbone. The name ’CSPDarknet53’ comes with the usage of Cross Stage Partial Networks (CSP) in it. CSP helps in dealing with the same gradients in large convolutional neural networks. This, in turn, helps reduce the number of parameters and FLOPs. Additionally, YOLOv5 uses the Path Aggregation Network (PANet) as the neck and YOLO detection heads [18]. Each of the suggested architectures varies in performance, depending on the size and the number of parameters used. The user can then utilize the architectures depending upon their use case scenario.

### 3.2. Overview of TPH-YOLOv5

TPH-YOLOv5, like our architecture, was designed for small object detection, specifically for drone-captured images. This architecture was inspired by YOLOv5 and modified to obtain robust results in scenarios where the size of the objects varies greatly and when there is the possibility of motion blur in the images due to motion. To solve these problems, TPH-YOLOv5 uses an additional prediction head, compared to the YOLOv5 architecture and Transformer Prediction Heads (TPHs), instead of the regular YOLO prediction heads. Additionally, the convolutional block attention model (CBAM) was integrated to find objects in densely packed areas. Furthermore, practical approaches such as data augmentation were also utilized during training to obtain better results.

The fundamental structural change in the TPH-YOLOv5 architecture is using Transformer prediction heads. These transformer prediction heads use the transformer encoder blocks from the vision transformer (ViT) architecture alongside some convolutional blocks and the CSP bottleneck blocks.

### 3.3. Transformers

**Figure 1:**
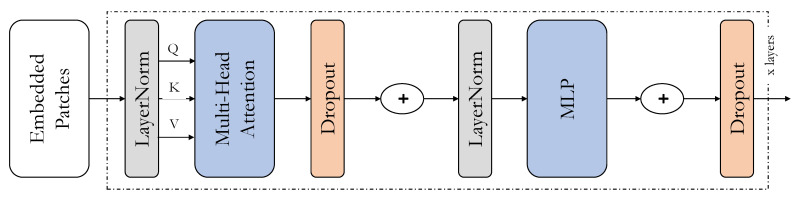
The architecture of the Vision Transformer.

As mentioned in the previous section, the Transformer encoder blocks have been used in the prediction heads of the TPH-YOlOv5 architecture. They have replaced the convolutional blocks, the CSP bottleneck blocks, and the backbone end in the original YOLOv5 with the transformer encoders. The transformer encoder block in the TPH-YOLOv5 seems to capture more global and contextual information. The major components of the transformer encoders are the multi-headed self-attention layer and the fully-connected layer. The architecture of the Vision Transformer is shown in Figure 1.

### 3.4. ConvMixer

**Figure 2:**
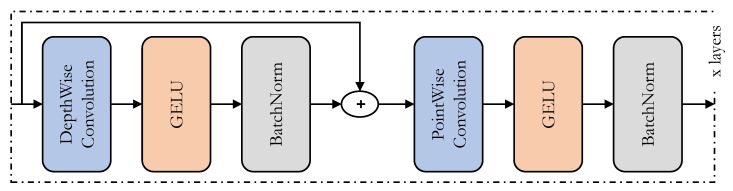
The architecture of the ConvMixer.

Amidst the extensive research on transformer architectures for computer vision applications, new architectures called mixers [48,50] were introduced to challenge the transformers. The main idea is to jumble up the data both spatially and in terms of channels. With sufficient data, these architectures perform on par with the transformers, with a smaller footprint in terms of computation time and resources. In our application, we utilize the ConvMixer architecture, shown in Figure 2, in place of the prediction heads in the YOLOv5 architecture. ConvMixer in the prediction head would help find the spatial and channel relationships within the features delivered to the prediction heads. Such relationships are found using the depthwise and pointwise convolutions in ConvMixer. The pointwise convolution in ConvMixer also enhances the capability of the prediction head to detect minutely small objects, as it deals with individual data-point-level information. Furthermore, unlike conventional convolutional neural networks, the ConvMixer sustains the input structure throughout the mixer layers to make it suitable for use in prediction heads of object-detection architecture. The overall architecture of the ConvMixer is also simple compared to the Transformer architecture.

### 3.5. Comparing Transformers and ConvMixers

The ConvMixer was originally designed to check whether the success of architectures such as transformers [15] and mixers [50] was due to the use of patches. Hence, the ConvMixer and vision transformers are similar in many respects. Both maintain the size and resolution of the input throughout all of the layers and do not perform downsampling at every instance. Both architectures try to find the one-to-one relationships between all of the inputs. To find these relationships, whereas the transformers utilize multi-headed self-attention, the ConvMixers utilize the depthwise and pointwise convolutions. The depthwise and pointwise convolutions mix the input instances depthwise and in terms of channels, respectively. The main difference between the transformers and ConvMixers is in terms of complexity. The self-attention in transformers is much more complex than the combination of depthwise and pointwise convolutions used in the ConvMixer architecture. Hence, the transformers require much more computing resources as compared to the ConvMixers. Thus, replacing the multi-headed self-attention-based transformers by ConvMixers is easily justifiable and we have proceeded with replacing the transformers in the prediction head of the TPH-YOLOv5 architecture with the ConvMixers.

### 3.6. Convolutional Block Attention Module (CBAM)

**Figure 3:**
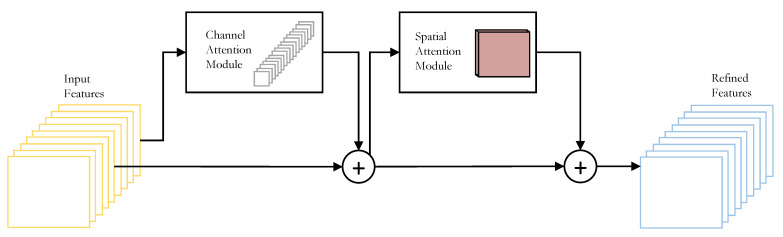
Overview of the CBAM module.

CBAM [49] is a simple, lightweight attention module that can be fused into most of the general CNN architectures. This module sequentially deduces the channel-wise and spatial attention maps, which are used to obtain the refined features by performing a product between the input features and the obtained attention maps. The general structure of CBAM can be seen in Figure 3. UAV-captured images could contain regions covering large areas in real life. Performing object detection in such scenarios could be tricky, so CBAM will help focus on the significant target objects.

### 3.7. Detailed System Overview

**Figure 4:**
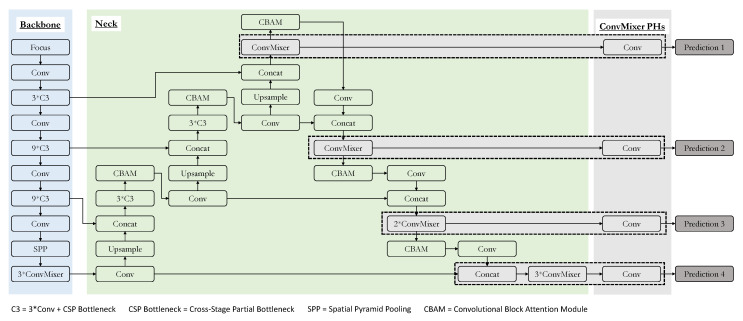
The architecture of the suggested model.

**Figure 5:**
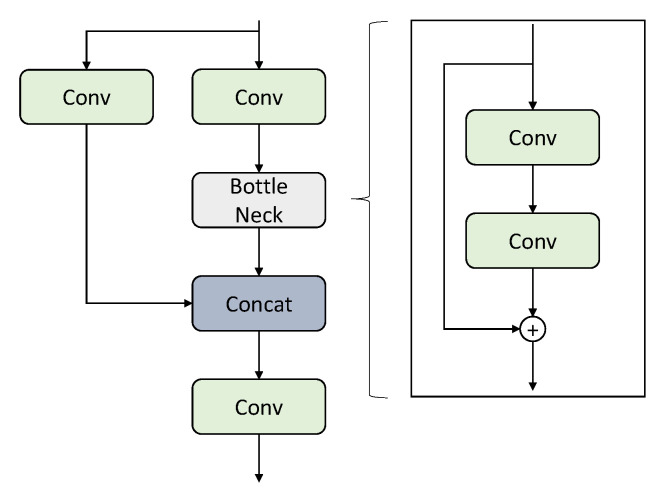
The structures of C3 block (**left**) and bottleneck (**right**).

The detailed framework of the suggested model is shown in Figure 4. The general architecture is similar to the baseline YOLOv5 model. The significant changes to the architecture come in the form of the prediction heads of the model. First, as opposed to the original YOLOv5 architecture, there is an additional prediction head to facilitate detection of small objects, making the resultant number of prediction heads sum up to four. The effectiveness of adding a prediction head to the existing three in the YOLOv5 model has been verified by the TPH-YOLOv5 architecture. Hence, we are just following suit. The next change to the prediction heads of the YOLOv5 is the usage of ConvMixers in them. TPH-YOLOv5 has attained quality results by using transformer encoders in the prediction heads. We have used ConvMixers in a similar way. The rationale for using the ConvMixers was justified in the previous section, and each of the prediction heads along with the final part of the backbone in the suggested architecture have ConvMixers. Finally, CBAM has been fused to different positions in the neck of the suggested architecture. This helps focus on the more significant use of the images when the field of view contains a large area in real life. The use of CBAM is also helpful, as seen in the TPHYOLOv5 architecture. In Figure 4, the C3 is the combination of three convolutional blocks along with the CSP bottleneck. The structure of the C3 block and the details of the CSP bottleneck are shown in Figure 5. To make the most of the available data and to make our model robust to simple variations, we have also adopted data augmentation strategies such as Mosaic and Mixup during training.

## 4. Experiments

In this section, we present the details of the implementation and experimental results of the suggested model. We substantially evaluated the suggested architecture alongside both the baseline YOLOv5 and TPH-YOLOv5 architectures using the VisDrone2021 dataset. Furthermore, we present the results of an ablation study to verify whether or not the performance gain is due to the suggested changes.

### 4.1. Dataset Description

**Figure 6:**
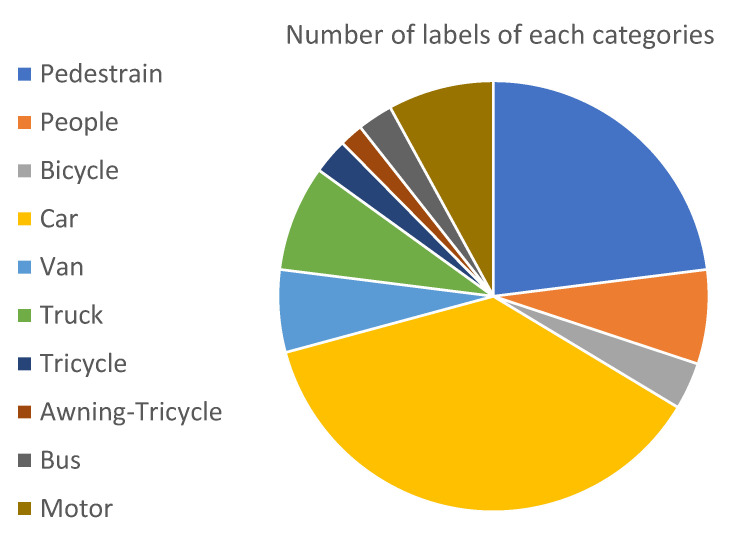
Pie chart describing the number of instances of labels for each category.

**Figure 7:**
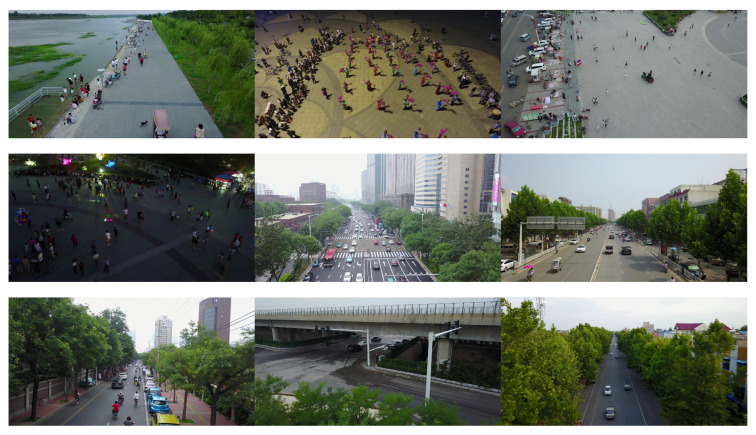
A few instances of the images in the VisDrone dataset.

We used the VisDrone-DET2021 dataset [51], which was used in the VisDrone-DET2021 Challenge, an ICCV 2021 workshop. The same dataset has been used previously in the VisDrone2019 [52] challenge. The goal is to predict the bounding boxes of objects of predefined classes with real-valued confidence. The dataset consists of 6471 images for training, 548 images for validation, and 3190 images for testing. There are ten object categories: pedestrian, person, bus, car, van, truck, bicycle, awning-tricycle, motorcycle, and tricycle. Figure 6 shows the distribution of the number of instances of the labels of each category in the VisDrone2021 dataset. Furthermore, Figure 7 shows a few instances of images in the VisDrone2021 dataset illustrating the variation of the dataset in terms size of the objects, background, number of objects in the frame, and motion of the objects.

### 4.2. Implementation Details

The code for the implementation of the suggested architecture was written in the PyTorch framework. The implementation and the detailed experimentation using the suggested model were performed on 4 NVIDIA RTX 2080 Ti GPUs, both during training and testing. Transfer learning was also utilized for better results, with the weights of the common parts in the baseline YOLOv5l model pretrained on the COCO dataset being extracted and used on the weights of the suggested architecture prior to starting the training. Additionally, the Adam optimizer [53] was employed during training with an initial learning rate of 3 × 10${}^{-4}$.

### 4.3. Evaluation Metrics

We mostly focus on two metrics: (i) $MeanAveragePrecision_{0.5}\left(mAP_{0.5}\right)$, and (ii) mean Average Precision (mAP). These evaluation metrics are expressed in the equation as follows:

(1) $$mAP_{0.5}=\frac{1}{n_{class}}\int_{0}^{1}P\left(R\right)dR$$

(2) $$\mathrm{mAP}=\frac{1}{N_{class}}\sum_{i=1}^{n_{class}}AP_{i}$$

where $N_{class}$ is the number classes. P and R are precision and recall, respectively, which are given by:

(3) $$P=\frac{TP}{TP+FP}$$

(4) $$R=\frac{TP}{TP+FN}$$

Here, TP means true positives, FP means false positives, and FN means false negatives.

### 4.4. Results

**Figure 8:**
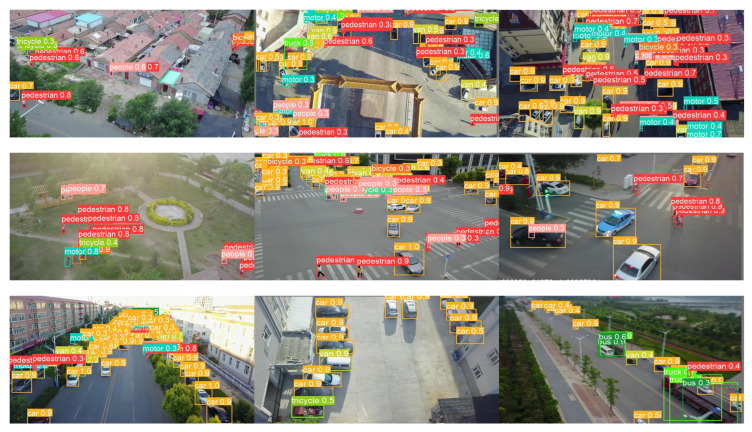
Visualization of some of the output of our suggested architecture on some of the test images of the VisDrone 2021 dataset.

We have evaluated the suggested network architecture in terms of precision, recall, $mAP_{0.5}$, and mAP, using the implementation details mentioned in Section 3.2. The suggested network architecture was compared against the TPH-YOLOV5 architecture [17] and various YOLOv5 architectures: YOLOv5n, YOLOv5s, YOLOv5m, YOLOv5l, and YOLOv5x. Table 1 shows the comparison results. The table shows that the proposed architecture performed better than the other models. Our model is 0.818% better in terms of precision, 0.788% better in terms of recall, 0.838% better in terms of $mAP_{0.5}$, and 0.46% better in terms of mAP scores than the second-best results, that is of TPH-YOLOv5. Figure 8 shows a visualization of some of the output of our architecture on some of the test images of the VisDrone 2021 dataset. The images used to visualize the output of the proposed model show variations in terms of the size of objects, viewing angle of the camera, background, and motion of the object. Figure 8 clearly shows that our suggested architecture adapts well to the size, background, and motion constraints that are present in the UAV imagery scenario.

**Figure 9:**
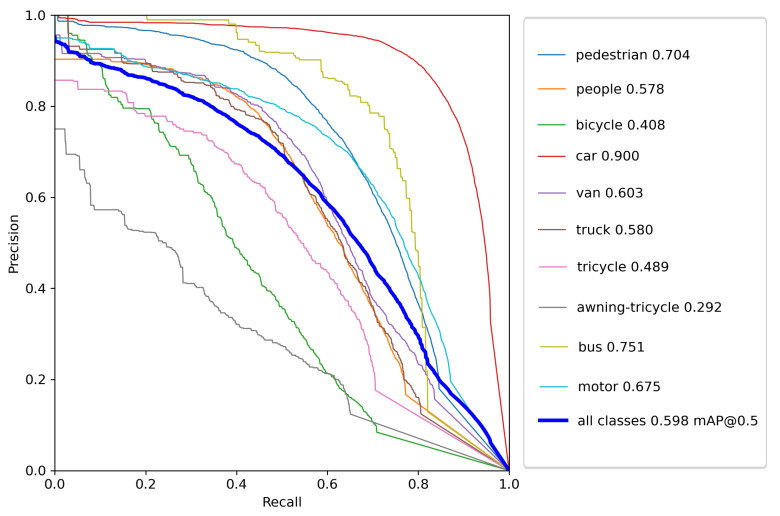
The P–R curve of the suggested model on the VisDrone2021 test set.

In Figure 9, the Precision-Recall (PR) curve is plotted for each class in the VisDrone 2021 dataset. The average precision for each class is indicated in the legend. From this curve, we can note that the suggested model performed the best while detecting the objects of the class ‘car’ and worst while detecting ‘awning-tricycle’. The average precision for the best classified and worst-classified classes ‘car’ and ‘awning-tricycle’ were 0.9 and 0.292, respectively. Furthermore, this conclusion is also backed by the Table 2, where the model performance on each if the individual classes has been presented. The main reason for this must be the number of instances available in the training dataset for each class, with the class ‘car’ occurring much more often than the class ‘awning-tricycle’. This can be verified from Figure 6. Additionally, while objects in the class ‘car’ are bigger and easily distinguishable, objects in ‘awning-tricycle’ tend to be quite small and could be as small as only a few pixels. Furthermore, object belonging to the class ‘awning-tricycle’ can also be confused with objects belonging to the class ‘tricycle’.

**Figure 10:**
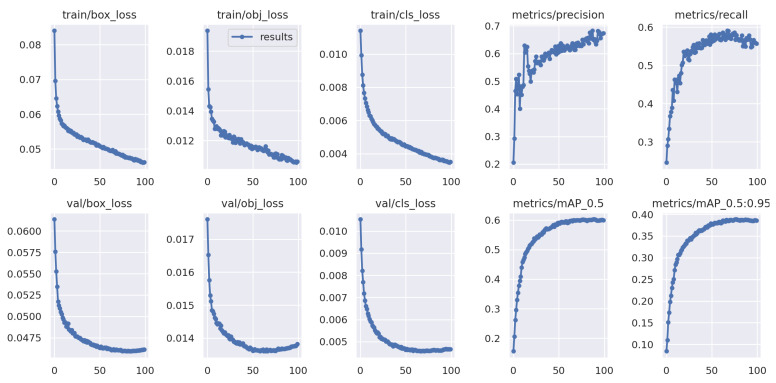
Visualization of various metrics (box loss, objective loss, class loss, precision, recall, $mAP_{0.5}$, $mAP_{0.5:0.95}$) with the number of epochs during training and validation.

**Figure 11:**
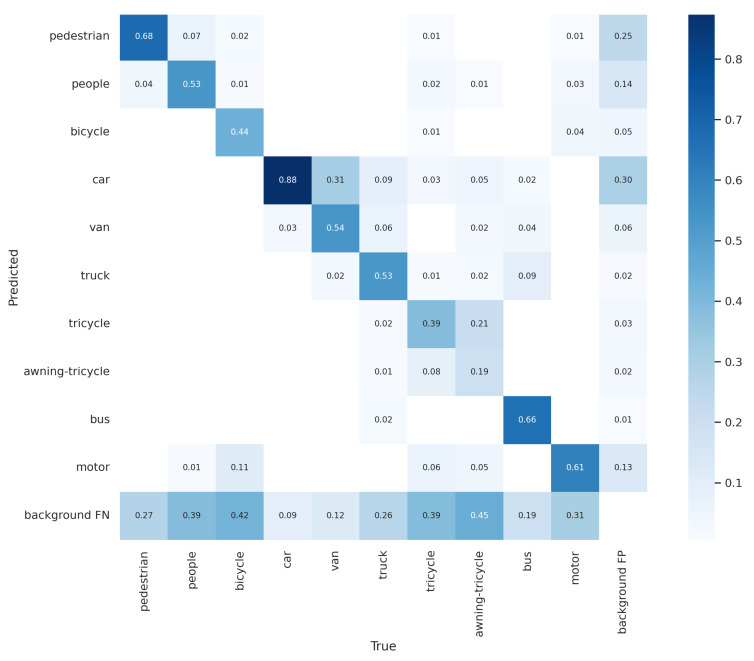
Confusion matrix for our method with the number of epochs during training and validation.

Figure 10 shows the progression of various metrics such as box loss, objective loss, and class loss during training and validation, and metrics such as precision, recall, $mAP_{0.5}$, and $mAP_{0.5:0.95}$, after each epoch. Figure 11 shows the confusion matrix of our model. The confusion matrix was constructed at an IoU threshold of 0.45 and confidence score of 0.25. Understanding the confusion matrix can help us understand the imperfections of our model. The major problem of the suggested model seems to be that many objects are not detected, i.e., they are considered the background. This mainly occurred with the classes ‘bicycle’, ‘tricycle’, and ‘awning-tricycle’, all of which are similar-looking and when captured from a high altitude using a drone are most likely to look much more similar to one another. Another reason could be the lower availability of the instances of these classes as compared to the other classes. The number of instances of the bicycle, tricycle, and awning-tricycle classes can be observed in Figure 6. Similarly, classes such as ‘car’, ‘van’, and ‘truck’, and also ‘bicycle’ and ‘tricycle’ are misclassified as one another. One of the reason for this misclassification is their similarity in appearance. These instances can be misclassified even when classified by a human.

### 4.5. Ablation Study

**Figure 12:**
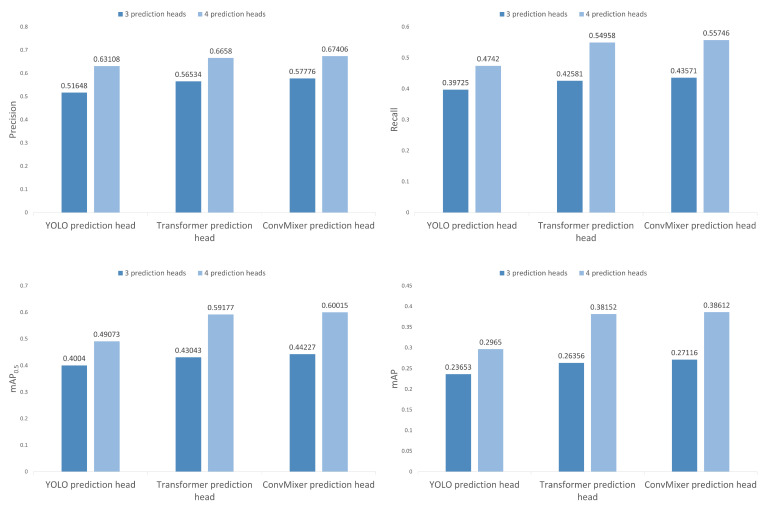
Comparision between the performance of the model while changing the number of prediction heads and the type of prediction head.

In this section, we present the results of an ablation study performed to understand the changes in the performance of object detection when there is variation in the number of prediction heads and the type of prediction heads used. For this, the number of prediction heads used was three and four, and the type of prediction heads used were the regular YOLO prediction head, transformer prediction head as used in TPHYOLO [17], and a ConvMixer [48] prediction head as used in our model. In Figure 12, the precision, recall, and $mAP_{0.5}$ and mAP are compared side by side in bar diagrams while making the abovementioned changes. From Figure 12, we can see massive improvements in all metrics no matter which type of prediction head is used when the number of prediction heads is increased from three to four. The average improvements in precision, recall, $mAP_{0.5}$, and mAP using each type of prediction head are 18.88%, 25.46%, 31.91%, and 37.5%, respectively. Furthermore, we can also see that the results when replacing the YOLO prediction head with the ConvMixer prediction head is better than when replacing them with Transformer prediction heads. This is the same no matter whether the number of prediction heads is three or four. The average improvement in precision, recall, $mAP_{0.5}$, and mAP when replacing the YOLO prediction heads with Transformer prediction heads are 7.48%, 11.54%, 14.04%, and 20.05%, respectively. On the other hand, the average improvements in precision, recall, $mAP_{0.5}$ and mAP when replacing the YOLO prediction heads with ConvMixer prediction heads are 9.33%, 13.61%, 16.37%, and 22.43%, respectively, which are at least 2% greater in terms of each metric as compared to when replaced by the Transformer prediction heads. These results support our claim that using ConvMixers as prediction heads of the YOLOv5 architecture helps in performance improvement for object detection in UAV imagery scenarios.

## 5. Conclusions

To deal with the problems introduced by UAV images during object detection, this paper presented a YOLOv5-like architecture with key changes such as the usage of ConvMixers on the prediction head and the provision of an additional prediction head to deal with smaller objects. The suggested architecture seems to deal with issues of UAV images much better compared with its predecessors such as YOLOv5 and TPH-YOLOv5. This seems to be because the depthwise convolution and the pointwise convolution in the ConvMixer of the prediction heads tend to mix the learned features spatially and in terms of channels to obtain the one-to-one relationships between each feature. Additionally, the added prediction head seems to make the architecture more robust against smaller-sized objects.

The effectiveness of the method has been verified using the popular VisDrone 2021 dataset, with the suggested model outperforming each of the compared architectures. However, the results are imperfect, and further improvements can still be made. For instance, objects are sometimes identified as background, and sometimes it is the other way around. Additionally, some similar-looking classes such as ‘car,’ ‘truck’, and ‘van’ and also ‘bicycle’ and ‘tricycle’ are being misclassified as one another.

In future work, the shortcomings of the paper can be further improved. Additionally, UAV devices focus on mobility and tend to have minimal compute resources. Hence, the algorithms to be run on these devices must require less computing time and resources. Further work can be done on designing more lightweight models or pruning the existing models to make them more suitable to be used in UAV scenarios. 

## Figures and Tables

**Table 1:** Comparison results between the suggested architecture and other methods.

Method	P (%)	R (%)	${mAP}_{0.5}$ (%)	mAP (%)
YOLOv5n	0.36189	0.28197	0.26161	0.13335
YOLOv5s	0.45251	0.3377	0.33179	0.18063
YOLOv5m	0.50072	0.37865	0.37837	0.21837
YOLOv5l	0.51648	0.39725	0.40004	0.23653
YOLOv5x	0.57061	0.39677	0.41358	0.24901
TPH-YOLOv5	0.66588	0.54958	0.59177	0.38152
Ours	**0.67406**	**0.55746**	**0.60015**	**0.38612**

**Table 2:** Model performance comparison on each of the individual classes.

Classes	P (%)	R (%)	${mAP}_{0.5}$ (%)	mAP (%)
All	0.638	0.570	0.598	0.389
Pedestrian	0.677	0.664	0.704	0.377
People	0.711	0.504	0.578	0.259
Bicycle	0.467	0.418	0.408	0.224
Car	0.766	0.887	0.900	0.679
Van	0.597	0.598	0.603	0.461
Truck	0.583	0.581	0.580	0.421
Tricycle	0.638	0.433	0.489	0.319
Awning-tricycle	0.483	0.265	0.292	0.203
Bus	0.734	0.737	0.751	0.588
Motor	0.725	0.611	0.675	0.361

## Data Availability

The dataset presented in this study has been made available to download by the corresponding authorities at http://aiskyeye.com/download/, (accessed on 5 July 2022). The copyright information is present at http://aiskyeye.com/data-protection/, (accessed on 1 October 2022), which mentions that the dataset can be used for academic purposes.

## Abbreviations

The following abbreviations are used in this manuscript:

UAV	Unmanned Aerial Vehicles
YOLO	You Only Look Once
CNN	Convolutional Neural Networks
CBAM	Convolutional Block Attention Module
PANet	Path Aggregation Network
TPH	Transformer Prediction head
GELU	Gaussian Error Linear Unit
HOG	Histogram of Oriented Gradients
SIFT	Sacle Invariant Feature Transform
FCOS	Fully Convolutional One-Stage
IoU	Intersection of Union
FSAF	Feature Selective Anchor Free Module
GFL	Generalized Focal Loss
DGQP	Distribution-Guided Quality Predictor
RCNN	Region Convolutional Neural Network
SPP	Spatial Pyramid Pooling
SSD	Single-Shot multibox Detector
CSPNet	Cross Stage Partial Network
CIoU	Complete Intersection of Union
